# A Colorimetric and Luminescent Dual-Modal Assay for Cu(II) Ion Detection Using an Iridium(III) Complex

**DOI:** 10.1371/journal.pone.0099930

**Published:** 2014-06-13

**Authors:** Dik-Lung Ma, Hong-Zhang He, Daniel Shiu-Hin Chan, Chun-Yuen Wong, Chung-Hang Leung

**Affiliations:** 1 Department of Chemistry, Hong Kong Baptist University, Kowloon Tong, Hong Kong, China; 2 Department of Biology and Chemistry, City University of Hong Kong, Tat Chee Avenue, Kowloon, Hong Kong SAR, People's Republic of China; 3 State Key Laboratory of Quality Research in Chinese Medicine, Institute of Chinese Medical Sciences, University of Macau, Macao, China; Griffith University, Australia

## Abstract

A novel iridium(III) complex-based chemosensor bearing the 5,6-bis(salicylideneimino)-1,10-phenanthroline ligand receptor was developed, which exhibited a highly sensitive and selective color change from colorless to yellow and a visible turn-off luminescence response upon the addition of Cu(II) ions. The interactions of this iridium(III) complex with Cu^2+^ ions and thirteen other cations have been investigated by UV-Vis absorption titration, emission titration, and ^1^H NMR titration.

## Introduction

The copper(II) ion plays a significant role in a number of physiological processes in living organisms, but is also an important environmental pollutant [1]. Aberrant levels of Cu^2+^ ions can result in oxidative stress, and has been linked with the development of Indian childhood cirrhosis, prion disease, Menkes disease, Parkinson's disease and Wilson disease [2]. The upper limit for the concentration of copper in drinking water has been recommended to be 2 ppm by the World Health Organization (WHO) [3]. A number of Cu^2+^-selective chemosensors that employ the chromogenic [4], [5], , fluorogenic [8], [9], [10], [11], [12], [13], [14], [15], or electrochemical [16], [17], [18] properties of molecules have been reported in the literature. However, these methods may require tedious sample pretreatment and/or multi-step synthetic procedures, or they may be limited by an unstable detection signal. Therefore, the development of sensitive and selective sensors for Cu^2+^ ions is of high interest [19].

The application of transition metal complexes as colorimetric and luminescent probes has recently attracted increasing attention [20], [21], [22], [23], [24], [25], [26], [27], [28], [29], [30] due to their notable advantages. Firstly, the absorptive and emissive behaviour of transition metal complexes can be sensitive to changes in the surrounding environment, allowing changes in analyte concentration to be transduced into an optical response [31], [32]. Secondly, metal complexes can possess significant Stokes shifts, allowing easy distinguishing of excitation and emission light [33], [34], [35], [36], [37], [38], [39], [40], [41], [42], [43], [44], [45], [46]. Third, the relatively long lifetimes of phosphorescent metal complexes compared to organic luminophores can allow interference from scattered light and short-lived background fluorescence to be reduced to a negligible level by use of time-resolved luminescence spectroscopy [47], [48]. Finally, the luminescence quantum yield of transition metal complexes can be enhanced by increased intersystem-crossing rates arising from strong spin-orbit interactions [49]. Among transition metal complexes, octahedral *d* ^6^ Ir(III) complexes have gained particular interest due to their decent thermal stability, intense luminescence at ambient temperature, and absorption or emission wavelengths across the entire visible light region that can be adjusted by modification of the auxiliary ligands [30], [50], [51].

**Figure 1:**
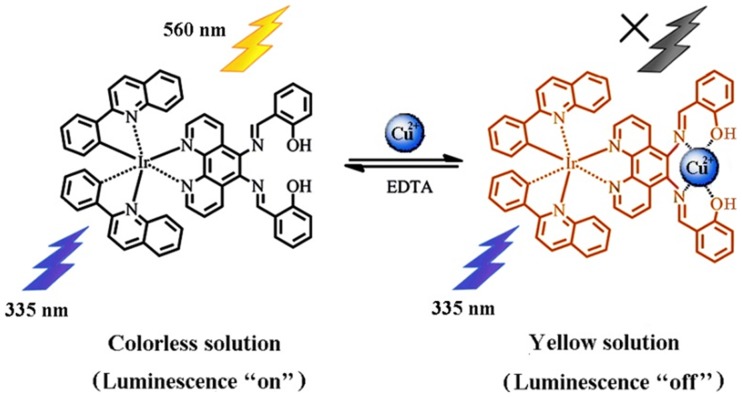
Chemical structure of [Ir(peq)_2_(sa2p)] (1) and proposed formation of 1-Cu^2+^ resulting in a colorimetric and luminescence response. The addition of EDTA restores the original state of the system.

**Figure 2:**
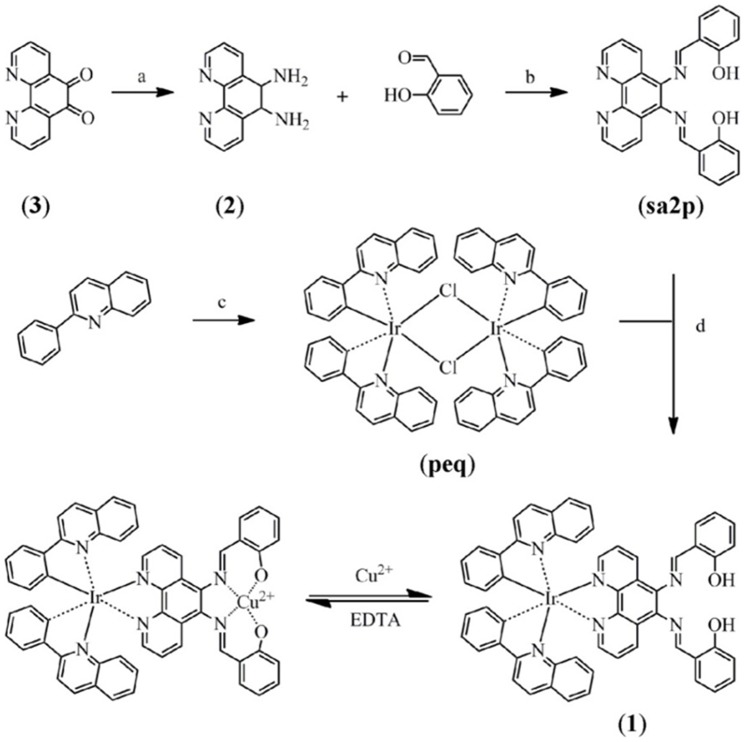
Synthetic pathway of 1. a) NH_2_OH·HCl, BaCO_3_, Pd/C, N_2_H_4_·H_2_O, reflux in EtOH; b) reflux in dry MeOH; c) stir in 2-ethoxyethanol at 100°C; (d) reflux in ethylene glycol.

A few iridium(III) complexes have been developed for Cu^2+^ detection, such as the phosphorescent cyclometalated iridium(III) complex containing the di(2-picolyl)-amine (DPA) copper ion receptor as reported by the group of Lippard, Nam and You [52], and the phosphorescent cyclometalated iridium(III) complex incorporating 3,9-dithia-6-azaundecane receptor by Hyun and co-workers [53]. In this work, we designed and synthesized a novel cyclometalated iridium(III) complex [Ir(peq)_2_(sa2p)] (denoted as **1**) containing two 2-phenylquinoline (peq) C∧N ligands and a single 5,6-bis(salicylideneimino)-1,10-phenanthroline (sa2p) tetradentate Schiff base receptor (Figure 1), which could function as both a colorimetric and luminescent chemosensor for Cu^2+^ detection. The synthetic pathway leading to the iridium(III) complex **1** is shown in Figure 2. In our design strategy, the interaction of the Cu^2+^ ion with the tetradentate Schiff base receptor can induce electron transfer from the metal center to the sa2p ligand, thereby influencing the photophysical behaviour of the iridium(III) complex. Detailed experimental procedures, characterization and photophysical properties of complex **1** are given in the ESI (Table S1 and Figure S1 in File S1).

## Experimental Section

### 2.1. Materials

1,10-Phenanthroline (99%), 2-phenylquinoline (99%), salicylic aldehyde (98%), hydrazine hydrate (79%) and hydroxylamine hydrochloride (98%) were purchased from Sigma Aldrich (St. Louis, MO, USA) and used as received. Iridium chloride hydrate (IrCl_3_·xH_2_O) was purchased from Precious Metals Online (Australia). All manipulations involving air-sensitive reagents were performed in an atmosphere of dry N_2_ gas. The solvents (diethyl ether, ethylene glycol monomethyl ether, ethylene glycol and acetonitrile) were purified by routine procedures and distilled under dry N_2_ before use. The solutions of metal ions were prepared from NaCl, KCl, CaCl_2_, MgSO_4_, FeCl_3_, Mn(NO_3_)_2_·6H_2_O, CoCl_2_·6H_2_O, NiCl_2_·6H_2_O, Zn(NO_3_)_2_, CdCl_2_, CuCl_2_·2H_2_O, HgCl_2_, AgNO_3_, Pb(NO_3_)_2_, respectively, and were dissolved in deionized water. Aqueous Tris-HCl (0.1 mol L^−1^) solution was used as buffer to keep pH value (pH = 7.0), and to maintain the ionic strength of all solutions in experiments.

### 2.2. Characterization

5,6-Bis(salicylideneimino)-1,10-phenanthroline (sa2p) ligand was first prepared via an established literature procedure [54]. The precursor complex [Ir_2_(peq)_4_Cl_2_] was synthesized according to the literature method [55], [56]. Complex **1** was prepared according to a modification of a previously reported procedure (Figure 2) [57]. ^1^H and ^13^C NMR were recorded on a Bruker Avance 400 spectrometer operating at 400 MHz (^1^H) and 100 MHz (^13^C). Mass spectra were obtained by using an Agilent 1100 Series LC/MSD or a JEOL JMS-600W mass spectrometer. Absorption and luminescence spectra were studied on a Cary 300 UV/Vis spectrophotometer and a PTI QM-4 spectrofluorometer (Photo Technology International, Birmingham, NJ), respectively.

#### 2.2.1. Synthesis of 1,10-phenanthroline-5,6-dione (3)

The ligand 1,10-phenanthroline-5,6-dione was prepared from a modification of the literature method [58]. To a stirring solution of concentrated H_2_SO_4_ (30 mL) in an ice bath, 1,10-phenanthroline (5.0 g, 23.8 mmol) was added. To this solution at 0–5°C, 2.5 g NaBr and 15 mL concentrated HNO_3_ were added slowly. The mixture was stirred at room temperature for 20 min, and was then refluxed for 1 h. After it was allowed to cool to room temperature, the solution was neutralized with 10% wt NaOH, and then filtered. The precipitate was dissolved in hot water and filtered when hot, followed by extraction with 200 mL CH_2_Cl_2_ three times. The organic phase was collected and after the removal of the solvent, the yellow solid was dried under vacuum. Yield: 3.4 g (68%). ^1^H NMR (400 MHz, CDCl_3_) δ 9.15–9.04 (m, 2H), 8.48 (dt, J = 12.6, 6.3 Hz, 2H), 7.57 (dt, J = 15.4, 7.7 Hz, 2H). ^13^C NMR (100 MHz, CDCl_3_) δ 178.67, 156.44, 152.91, 137.34, 128.07, 125.64. HRMS (ESI, m/z): [M + H]^+^ calcd for C_12_H_6_N_2_O_2_, 210.0429; found: 210.0526.

#### 2.2.2. Synthesis of 5,6-diamine-1,10-phenanthroline (2)

The synthesis of 1,10-phenanthroline-5,6-diamine can be accomplished in two steps [58]. A mixture of 1,10-phenanthroline-5,6-dione (0.42 g, 2.0 mmol), NH_2_OH·HCl (0.5 g, 7.2 mmol) and BaCO_3_ (3.0 g) was refluxed in ethanol (30 mL) for 17 h. After filtration, the residue was treated with 0.2 M HCl (40 mL), stirred for 30 min and filtered. The yellow solid was washed successively with H_2_O, ethanol and diethyl ether, and finally dried under vacuum. Yield of 5,6-dioxime-1,10-phenanthroline: 0.46 g (94%). The dioxime was used a starting material for the synthesis of the diamine without future purification. A mixture of 5,6-dioxime-1,10-phenanthroline (0.8 g) and Pd/C (10%, 1.0 g) in ethanol (200 mL) was purged with N_2_ and heated to reflux. N_2_H_4_·H_2_O (7 mL) and ethanol (30 mL) were added over a period of 1 h. The solution was refluxed for 24 h and filtered, and the solid was washed with boiling H_2_O (150 mL) five times. The filtrate was dried under vacuum, triturated in 60 mL H_2_O and kept at 4°C overnight. The residue was filtered and washed with cold H_2_O, and dried under vacuum. Yield: 0.48 g (67%). ^1^H NMR (400 MHz, CDCl_3_) δ 8.76 (dd, J = 4.2, 1.5 Hz, 2H), 8.50 (dd, J = 8.5, 1.5 Hz, 2H), 7.62 (dd, J = 8.4, 4.2 Hz, 2H). ^13^C NMR (100 MHz, CDCl_3_) δ 145.27, 140.61, 129.42, 123.15, 122.70, 122.58. HRMS (ESI, m/z): [M+H]^+^ calcd for C_12_H_12_N_4_, 212.1062; found: 213.1034.

#### 2.2.3. Synthesis of 5,6-bis(salicylideneimino)-1,10-phenanthroline (sa2p)

5,6-Diimino-1,10-phenanthroline (0.21 g, 1 mmol) and salicylaldehyde (0.25 g, 2.1 mmol) were dissolved in absolute methanol (50 mL) and refluxed for 0.5 h. The precipitate was filtered off and washed with ethanol and water. The product was obtained as a yellow solid. Yield 0.24 g (58%). ^1^H NMR (400 MHz, CDCl_3_) δ 13.54 (s, 2H), 9.04 (dd, J = 4.3, 1.7 Hz, 2H), 8.92 (dd, J = 8.1, 1.7 Hz, 2H), 8.18–8.09 (m, 2H), 7.84 (m, 6H), 7.00 (m, J = 8.8 Hz, 4H). ^13^C NMR (100 MHz, CDCl_3_) δ 158.95, 151.17, 147.48, 144.75, 143.28, 136.54, 135.70, 129.48, 124.81, 123.17, 121.08, 115.76. HRMS (ESI, m/z): [M+H]^+^ calcd for C_26_H_18_N_4_O_2_, 418.1430; found: 419.3359.

#### 2.2.4. Synthesis of [Ir_2_(peq)_4_Cl_2_] dimer

2-Phenylquinoline (0.20 g, 0.98 mmol) was dissolved in 2-ethoxyethanol (15 mL) in a 50 mL round-bottom flask. Iridium trichloride hydrate (0.15 g, 0.5 mmol) and 5.0 mL of water were then added to the flask. The mixture was stirred under nitrogen at 100°C for 24 h and was cooled to room temperature. The precipitate was collected, washed with water and dried under vacuum to give the cyclometalated [Ir(peq)_2_Cl_2_] dimer.

#### 2.2.5. Synthesis of [Ir(peq)_2_(sa2p)] (1)

A suspension of the dimer [Ir_2_(peq)_4_Cl_2_] (127.22 mg, 0.5 mmol) and 5,6-bis(salicylideneimino)-1,10-phenanthroline (sa2p) (200.71 mg, 0.22 mmol) in ethylene glycol was refluxed overnight under a nitrogen atmosphere. The resulting solution was then allowed to cool to room temperature and 10 mL of H_2_O was added. The solution was extracted three times with diethyl ether. To the filtrate, an aqueous solution of ammonium hexafluorophosphate (excess) was added and the filtrate was reduced in volume by rotary evaporation until precipitation of the crude product occurred. The precipitate was then filtered and washed with several portions of water (2×50 mL) followed by diethyl ether (2×50 mL). The product was recrystallized by acetonitrile/diethyl ether vapor diffusion to yield the titled compound as an orange solid. Yield 214.04 mg (21%). ^1^H NMR (400 MHz, acetone) δ 13.29 (s, 2H), 12.03 (s, 2H), 9.12 (dd, *J* = 48.1, 8.2 Hz, 2H), 8.70 (t, *J* = 5.1 Hz, 2H), 8.52 (dd, *J* = 32.8, 8.9 Hz, 4H), 8.31 (dd, *J* = 7.7, 2.6 Hz, 2H), 8.19–8.08 (m, 2H), 7.99 (d, *J* = 7.8 Hz, 2H), 7.83–7.72 (m, 2H), 7.69–7.21 (m, 8H), 7.11–6.79 (m, 6H), 6.68 (dd, *J* = 7.6, 2.4 Hz, 2H), 5.33 (s, 2H); ^13^C NMR (100 MHz, DMSO) δ 161.17, 157.75, 150.11, 149.89, 149.05, 147.73, 146.00, 139.31, 137.37, 136.47, 135.22, 132.54, 129.76, 129.22, 128.51, 128.25, 127.49, 127.42, 127.33, 126.28, 121.25, 119.51, 119.24, 116.74. HRMS (ESI, m/z): [M+H]^+^ calcd for C_56_H_38_IrN_6_O_2_, 1019.2685; found: 1019.3433. Anal. calcd for C_56_H_38_IrN_6_O_2_PF_6_: C, 57.78; H, 3.29; N, 7.22; found: C, 57.66; H, 3.13; N, 7.01.

#### 2.2.6. Photophysical measurement

Emission spectra and lifetime measurements for **1** were performed on PTI QM-4 spectrofluorometer (Nitrogen laser: pulse output 335 nm) fitted with a 400 nm filter. Error limits were estimated: λ (±1 nm); τ (±10%); φ (±10%). All solvents used for the lifetime measurements were degassed using three cycles of freeze-vac-thaw.

Luminescence quantum yields were determined using the method of Demas and Crosby [59] [Ru(bpy)_3_][PF_6_]_2_ in degassed acetonitrile as a standard reference solution (*Φ*_r_ = 0.062).

#### 2.2.7. Calculation of binding constants

The binding constants (*K*) were determined from the Benesi−Hildebrand plot [60].

## Results and Discussion

### 3.1 UV-Vis absorption spectroscopy

**Figure 3:**
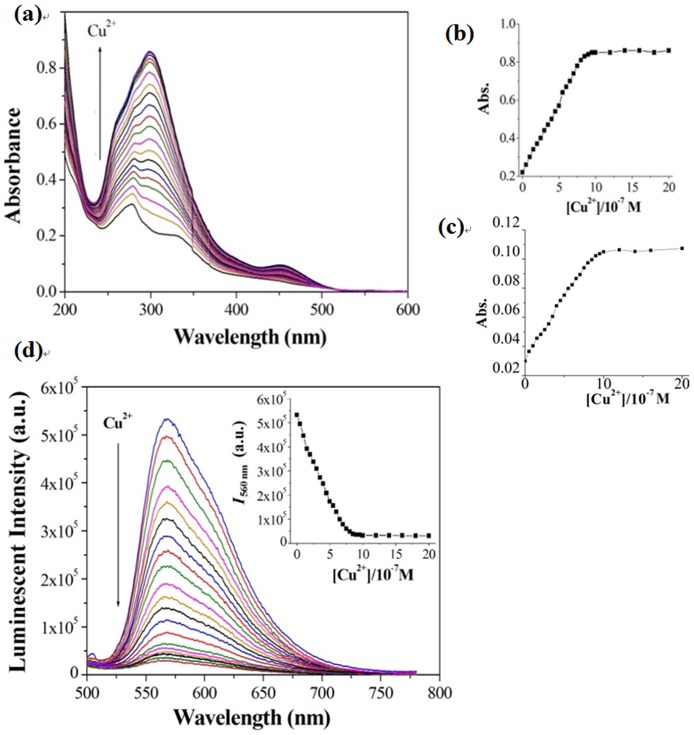
UV-Vis absorption spectra (a) of 1 (1 µM) in CH_3_CN solution with various amounts of Cu^2+^ ions (0–2 µM). (b) The relationship between absorbance of **1** at 290 nm *vs.* [Cu^2+^]. (c) The relationship between absorbance of **1** at 462 nm *vs.* [Cu^2+^]. (d) Luminescence spectra of **1** (1 µM) with various amounts of Cu^2+^ ions (0–1 µM) in CH_3_CN solution. Inset: emission of **1** at 560 nm *vs.* [Cu^2+^]. ions. λ_ex_ = 355 nm.

We first performed a UV-Vis absorption titration experiment to investigate whether complex **1** could be used as a colorimetric sensor for Cu^2+^ ions. Encouragingly, new absorption bands at 290 and 462 nm appeared when Cu^2+^ ions were added to a solution of complex **1** in CH_3_CN, which was accompanied by a color change of the solution from colorless to yellow (Figure 3a). The absorption band at 290 nm in the UV-Vis spectrum of complex **1** might originate from the allowed ^1^(π-π*) transitions of the C∧N ligand, while the weak absorption peak at 462 nm might arise from spin-forbidden ^3^MLCT transitions [61]. The absorbance intensities of the solution were increased by up to *ca.* 4.5-fold at 290 nm (Figure 3b) and 3.5-fold at 462 nm (Figure 3c) at saturating concentrations of Cu^2+^ ions. Importantly, the color change of the solution occurred within 10 s upon the addition of Cu^2+^ ions, indicating that **1** can serve as a simple and rapid ‘naked-eye’ indicator for Cu^2+^ ions (Figure S2a).

### 3.2 Luminescence response of complex 1 to Cu^2+^

**Figure 4:**
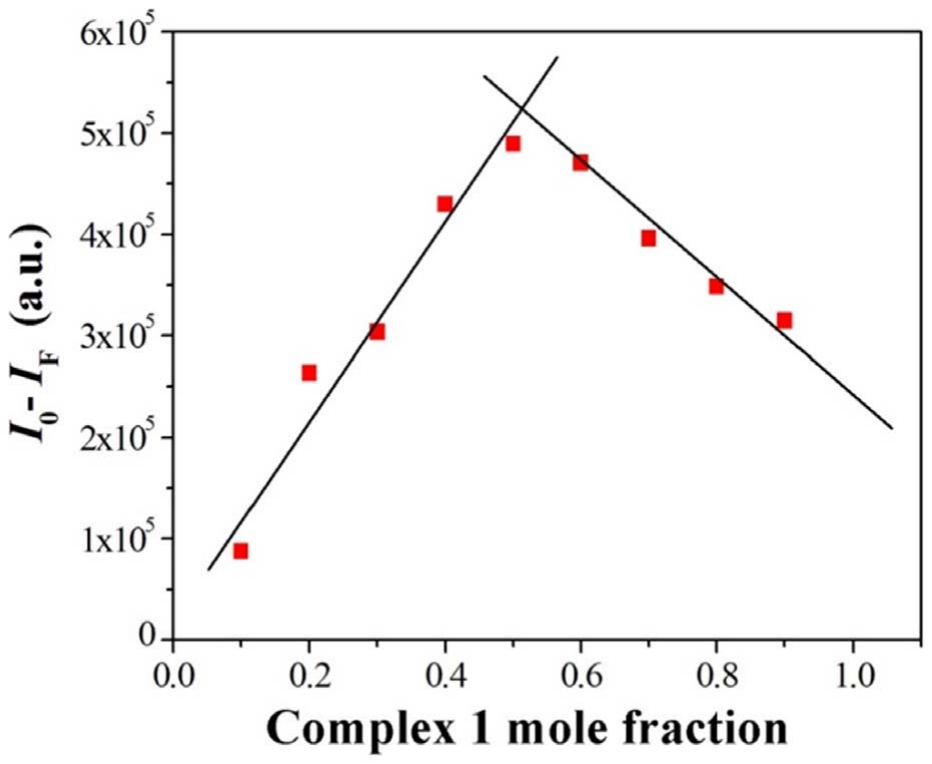
Job's plot analysis of luminescence titration data for 1 in CH_3_CN solution. The total concentration of 1 and Cu^2+^ is 1 µM. λ_ex_ = 355 nm, λ_em_ = 560 nm.

Emission spectroscopy offers the advantage of greater sensitivity towards small changes that affect the electronic properties of ligand receptors [62]. In CH_3_CN solution, complex **1** showed an intense orange emission at 560 nm with a quantum yield of 0.39 (Table S1 in File S1). Interestingly, a significant decrease of the luminescent intensity of **1** was observed with increasing concentration of Cu^2+^ ions, with nearly complete quenching (*φ* = 0.0031) exhibited at 1 equivalent of Cu^2+^ ions (Figure 3d and Figure S2b in File S1). The emission lifetime monitored at 560 nm in CH_3_CN solution at 25°C was measured to be 4.8 *µ*s. This long lifetime suggests that the excited states of the iridium(III) complex **1** have triplet character (^3^MLCT), resulting in phosphorescence emission [63]. In addition, a linear relationship (*R* ^2^ = 0.9863) between the luminescence intensity of **1** and the concentration of Cu^2+^ ions over the range of 1.0–8.0×10^−7^ M was observed (Figure S3 in File S1). The detection limit as defined by International Union of Pure and Applied Chemistry (IUPAC, detection limit = 3 Sb/m) was 2.26×10^−8^ M, which is lower than the acceptable value mandated for the concentration of copper in drinking water by the WHO and the US Environmental Protection Agency (EPA). Moreover, Job's plot analysis of the luminescence titration data revealed a maximum in quenching intensity at 0.5 mole fraction of **1**, indicating a 1: 1 stoichiometry between Cu^2+^ ions and **1** (Figure 4). On the basis of this stoichiometry, the binding constant value (*K*) calculated from the emission titration data was 4.8×10^4^ M^−1^ according to the Stern-Volmer equation [64].

### 3.3 ^1^H NMR titration experiments

**Figure 5:**
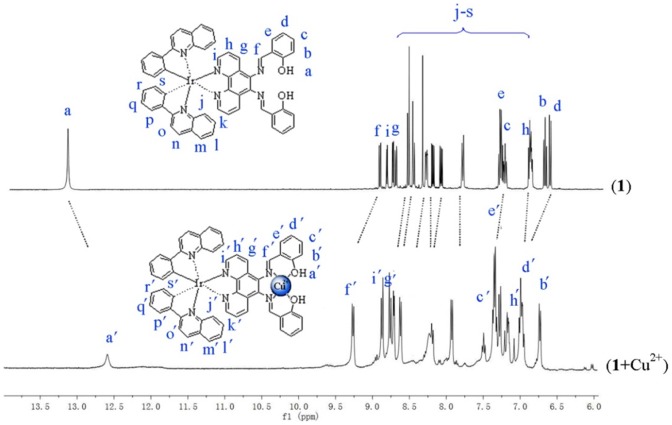
The proposed structure for 1-Cu^2+^, and ^1^H NMR spectra of 1 (5 mM) and CuCl_2_ (5 mM) in DMSO-*d*_6_.

^1^H NMR titration of **1** and 1-Cu^2+^ in DMSO-*d*_6_ was performed to determine the complexation mode of **1** to Cu^2+^ ions. The results showed several significant spectral changes in the ^1^H NMR spectra of **1** upon complexation with Cu^2+^ ions (Figure 5). For the aliphatic region, the peak for H_f_ on the receptor sa2p underwent a downfield shift of 0.52 ppm (from 8.75 to 9.27 ppm), suggesting that the Cu^2+^ ion is bound by the nitrogen atom of sa2p [65]. Additionally, the peak for the phenolic proton H_a_ is shifted from 13.24 to 12.65 ppm. The spectral changes observed are consistent with the putative binding of the Cu^2+^ ions to sa2p *via* coordination to two nitrogen atoms and two phenol groups.

### 3.4 Response of complex 1 to various metal ions

**Figure 6:**
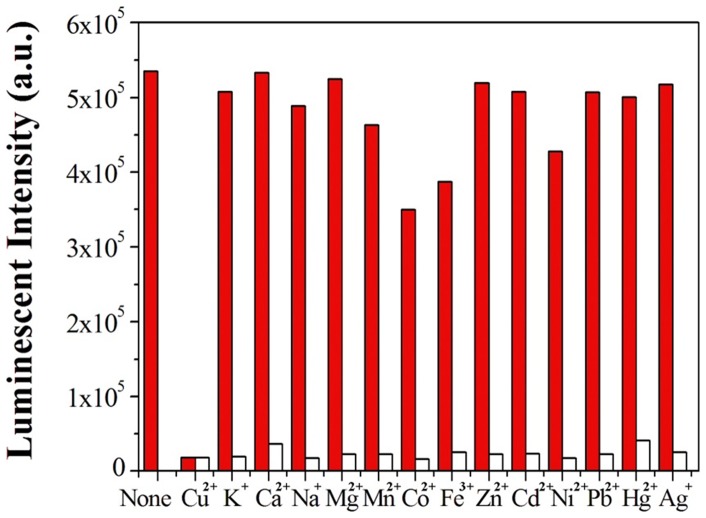
Red bars: luminescent emission response of 1 (1.0×10^−6^ M) at 560 nm in the presence of Cu^2+^ (1.0×10^−6^ M) or various other cations (1.0×10^−4^ M) in CH_3_CN solution. White bars: luminescent response of 1 at 560 nm in the presence of both Cu^2+^ (1.0×10^−6^ M) and other 13 cations (1.0×10^−4^ M). λ_ex_ = 355 nm.

**Figure 7:**
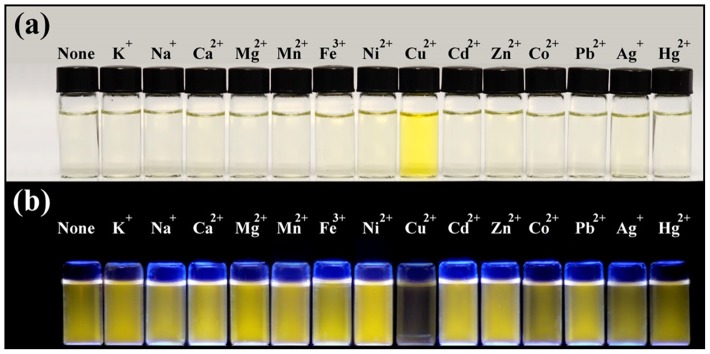
Photograph images of complex 1 (2 µM) in the presence of various metal ions (2 equivalents) in CH_3_CN solution under (a) white light or (b) UV irradiation.

We next investigated the luminescence responses of **1** to thirteen other cations in order to determine the selectivity of the iridium(III) complex for Cu^2+^ ions. At 1.0×10^−6^ M of Cu^2+^ ions, the luminescence intensity of complex **1** was quenched by 99.2%. On the other hand, the luminescence of complex **1** was not significantly affected in the presence of 1.0×10^−4^ M of K^+^, Na^+^, Mg^2+^, Ca^2+^, Cd^2+^, Fe^3+^, Pb^2+^, Ag^+^ and Hg^2+^, while 1.0×10^−4^ M of Mn^2+^, Co^2+^, Zn^2+^ and Ni^2+^ only resulted in quenching intensities of 13.4–22.6% (red bars in Figure 6). These results demonstrate that complex **1** is selective for Cu^2+^ ions over 100-fold excess of other cations. In order to evaluate the robustness of the system, competition experiments were performed in which both Cu^2+^ ions (1.0×10^−6^ M) and 100-fold excess of the other metal ions were simultaneously added to complex **1** (white bars in Figure 6). The results showed that the quenching of luminescence intensity of complex **1** by Cu^2+^ ions was not affected by the presence of the thirteen other cations. The selectivity of complex **1** was also confirmed by UV-Vis absorption spectroscopy, where only Cu^2+^ ions was able to induce significant changes in the absorption spectrum of **1** (Figure S4 in File S1). The selectivity of complex **1** for Cu^2+^ ions could be visually observed by the naked eye (Figure 7a) or under UV irradiation (Figure 7b). Thus, complex **1** could be potentially utilised as a simple optical chemosensor for the selective detection of Cu^2+^ ions.

### 3.5 Regeneration efficiency of the sensing system

**Figure 8:**
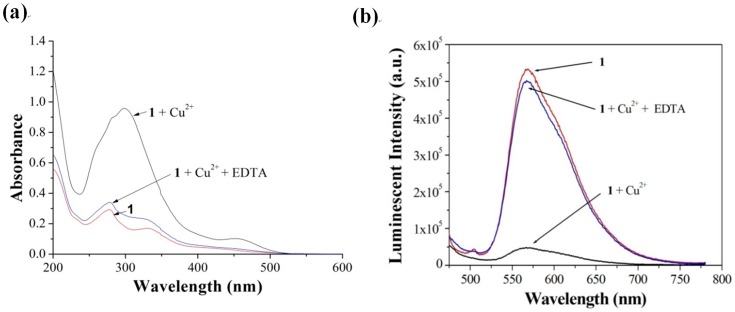
Absorption spectra (a) and luminescence emission spectra (b) of complex 1 (1 µM). Complex **1**/Cu^2+^([1] = 1 µM, [Cu^2+^] = 10 µM), and complex **1**/Cu^2+^/EDTA in CH_3_CN solution ([1] = 1 µM, [Cu^2+^] = 10 µM, [EDTA] = 20 µM).

Reusability is an important consideration for practical chemosensors. When ethylenediaminetetraacetic acid (EDTA) (20 µM) was introduced into a solution containing **1** (1 µM) and Cu^2+^ ions (10 µM), the color of the solution changed from yellow to colorless, with an absorbance increase that was only 8.6% that of the Cu^2+^-treated system (Figure 8a). Additionally, 89% of the original luminescence intensity of complex **1** was restored (Figure 8b). These results indicate that the association of complex **1** with Cu^2+^ ions is reversible, and that complex **1** could be used for repetitive Cu^2+^ ion sensing applications.

## Conclusion

In conclusion, we report a new iridium(III) complex **1** bearing the 5,6-bis(salicylideneimino)-1,10-phenanthroline ligand as a Cu^2+^-selective colorimetric and luminescent chemosensor, which represents, to our knowledge, one of the relatively few examples of dual colorimetric and luminescent iridium(III)-based Cu^2+^ ion sensors reported in the literature. A highly sensitive and selective color change from colorless to yellow and luminescent quenching effect were observed upon addition of Cu^2+^ ions to a solution of complex **1**. We believe that the novel iridium(III) complex **1** developed in this work can form the basis of naked-eye Cu^2+^ ions sensors for practical use.

## Supporting Information

File S1 Contains Table S1, Photophysical properties of complex 1 in CH_3_CN at 298 K. Figure S1, UV/Vis absorption spectrum of complex 1 (1 µM) in CH_3_CN solution at 298 K. Figure S2, White light (a) and UV light photograph images (b) of 1 (2 µM) in the presence of different concentrations of Cu^2+^ ions (0–10 µM) in CH_3_CN solution. Figure S3, Curve of luminescence intensity of 1 (1 µM) at 560 nm versus concentration of Cu^2+^ ions in CH_3_CN solution. λ_ex_ = 355 nm. Figure S4, UV-Vis absorption spectra of 1 (1 µM) in the presence of Cu^2+^ ion and 2 equivalents of thirteen other metal ions in CH_3_CN solution.
(DOCX)
