# Supplementary material for: A Colorimetric and Luminescent Dual-Modal Assay for Cu(II) Ion Detection Using an Iridium(III) Complex
Source: PLoS One. 2014 Jun 13;9(6):e99930. doi: 10.1371/journal.pone.0099930 (PMC4057321; doi:10.1371/journal.pone.0099930)
Supplement: File S1 — Contains Table S1, Photophysical properties of complex 1 in CH3CN at 298 K. Figure S1, UV/Vis absorption spectrum of complex 1 (1 µM) in CH3CN solution at 298 K. Figure S2, White light (a) and UV light photograph images (b) of 1 (2 µM) in the presence of different concentrations of Cu2+ ions (0–10 µM) in CH3CN solution. Figure S3, Curve of luminescence intensity of 1 (1 µM) at 560 nm versus concentration of Cu2+ ions in CH3CN solution. λex = 355 nm. Figure S4, UV-Vis absorption spectra of 1 (1 µM) in the presence of Cu2+ ion and 2 equivalents of thirteen other metal ions in CH3CN solution. (DOCX) [file pone.0099930.s001.docx]

**Electronic Supporting Information**

A colorimetric and luminescent dual-modal assay for Cu(II) ion detection using an iridium(III) complex

Dik-Lung Ma,*^*a^* Hong-Zhang He,*^a^* Daniel Shiu-Hin Chan,*^a^* Chun-Yuen Wong^b^, and

Chung-Hang Leung^c^

^a^ Department of Chemistry, Hong Kong Baptist University, Kowloon Tong, Hong Kong, China.

^b^ Department of Biology and Chemistry, City University of Hong Kong, Tat Chee Avenue, Kowloon, Hong Kong SAR, People's Republic of China

^c^ State Key Laboratory of Quality Research in Chinese Medicine, Institute of Chinese Medical Sciences, University of Macau, Macao, China.

* To whom correspondence should be addressed. Dik-Lung Ma. Tel: (+852)3411-7075; Fax: (+852)3411-7348; Email: edmondma@hkbu.edu.hk.

**Table S1.** Photophysical properties of complex **1** in CH_3_CN at 298 K.

| Complex | UV-Vis absorption | Emission | |
| --- | --- | --- | --- |
|  | λ_abs_ [nm] (*ε* [dm^3^mol^–1^cm^–1^]) | λ_em_ [nm] (τ [µs]) | Quantum yield *Φ* |
| **1** | 335 (2.23 × 10^4^), 276 (1.68 × 10^4^) | 560 (4.80) | 0.39 |

**
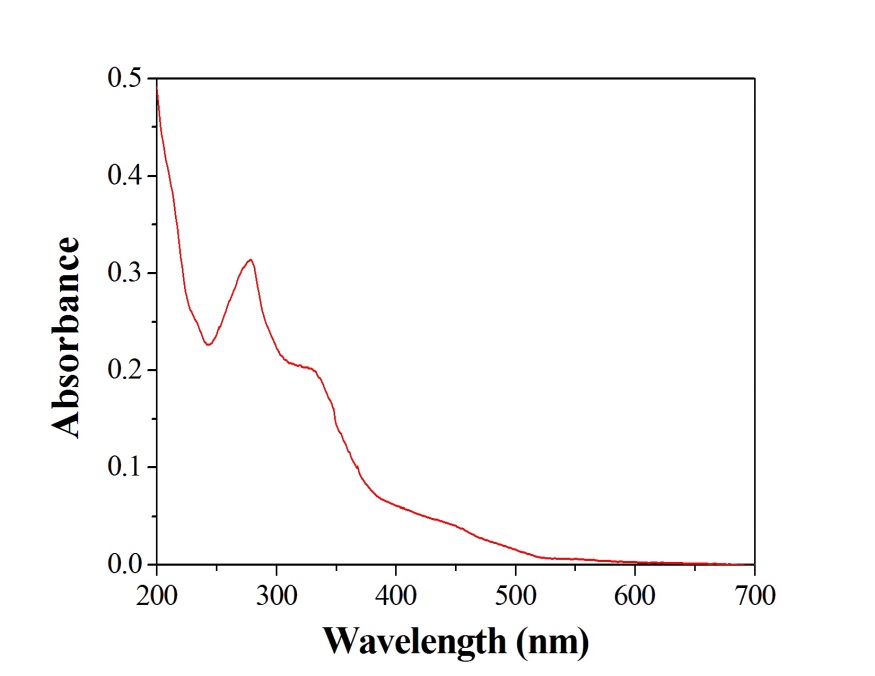
**

**Figure S1** UV/Vis absorption spectrum of complex **1** (1 µM) in CH_3_CN solution at 298 K.


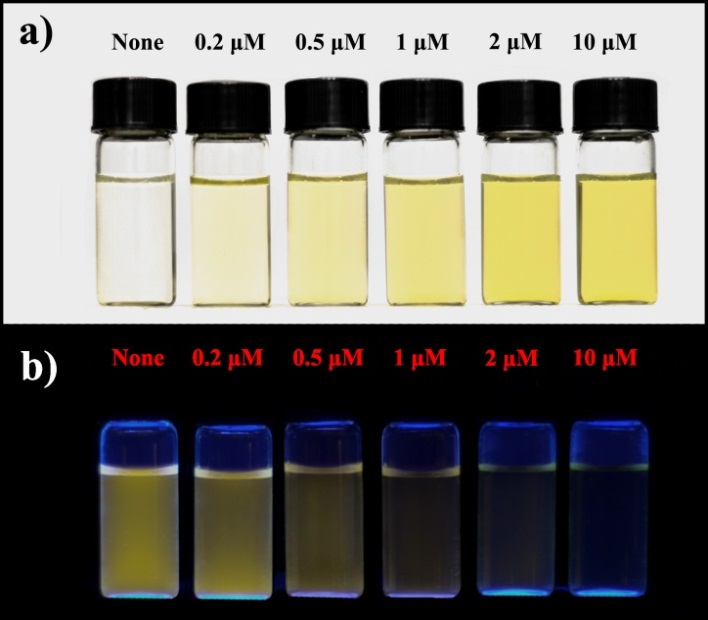


**Figure S2** White light (a) and UV light photograph images (b) of **1** (2 μM) in the presence of different concentrations of Cu^2+^ ions (0–10 μM) in CH_3_CN solution.


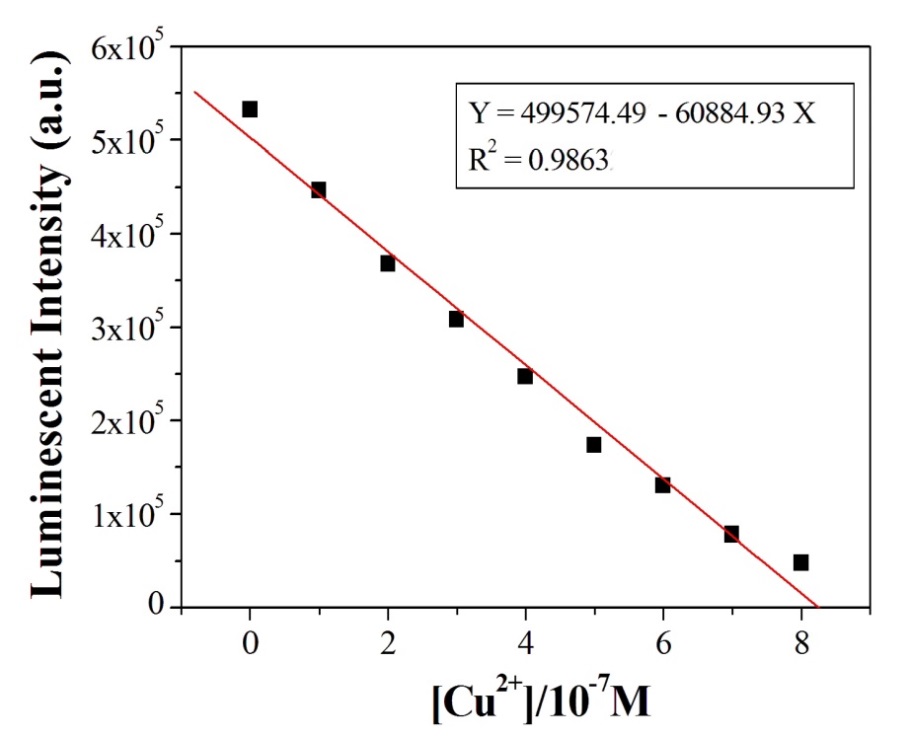


**Figure S3** Curve of luminescence intensity of **1** (1 μM) at 560 nm versus concentration of Cu^2+^ ions in CH_3_CN solution. λ_ex_ = 355 nm.


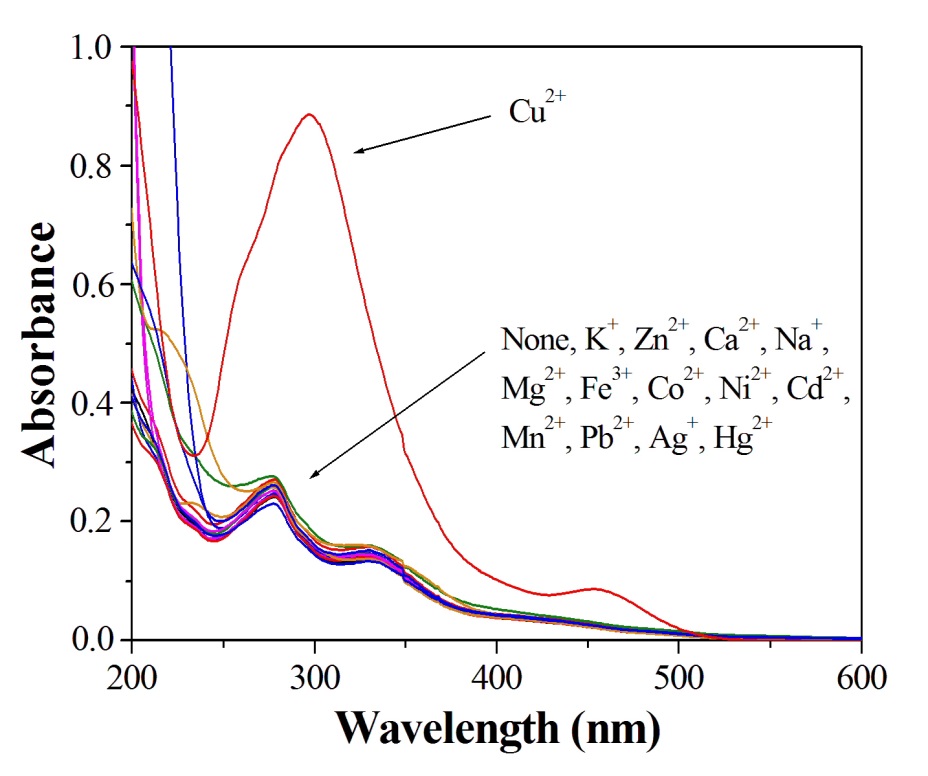


**Figure S4** UV-Vis absorption spectra of **1** (1 μM) in the presence of Cu^2+^ ion and 2 equivalents of thirteen other metal ions in CH_3_CN solution.
